# Effect of bibliotherapy on reducing anxiety among pre-operative patients in Gujarat, India

**DOI:** 10.6026/97320630018692

**Published:** 2022-08-31

**Authors:** N Sivasubramanian, Jaimin Vrajeshbhai Jaimin Vrajeshbhai, B Mahalakshmi, KJ Shaijo, Bhasara Kalpana Ramji

**Affiliations:** 1 Nootan College of Nursing, Sankalchand Patel University, Visnagar, Gujarat - 384315, India

**Keywords:** Bibliotherapy, Anxiety, Pre-operative patients

## Abstract

Numerous studies have shown that patients can have preoperative anxiety in mild, moderate, or severe forms. A supplemental tool to a disease's clinical treatment is bibliotherapy. This approach includes the core ideas of cognitive behavioral therapy and offers exercises meant to assist readers in overcoming unpleasant emotions. Therefore, it is of interest to evaluate how well bibliotherapy reduced pre-operative patients' anxiety. For the experimental group (30) and control group (30), a sample of 60 preoperative patients who had been determined to have considerable levels of anxiety was chosen. The Hamilton Anxiety Rating Scale is used to measure patient anxiety. Prior to surgery, bibliotherapy was given to the experimental group's sample twice daily for around 20 minutes. No intervention was given to the control group. The study's findings showed that the experimental group's mean percentage anxiety score at the pre-test was 80.10 percent, whereas the control group's mean percentage anxiety score was 85.66 percent. After the test, the experimental group's mean anxiety score was 50.66 percent, while the control group's mean anxiety score was 83.20 percent. It is evident that bibliotherapy was successful in lowering pre-operative patients' anxiety levels. Nurses can utilize this non-pharmacological technique to help patients feel less anxious about surgery and experience fewer post-operative problems.

## Background:

Anxiety is described by the American Psychological Association (APA) as emotions of tension, apprehension, uneasiness, fear, discomfort, and elevated autonomic activity that might range in intensity due to a perception of impending danger, a dangerous occurrence, or something unknown [1]. Surgeons and anaesthesiologists should be concerned about preoperative anxiety. Anxiety before surgery might be related to both anaesthesia and operation. Many patients experience anxiety at varying degrees of intensity [2]. According to certain research, psychological elements like fear and anxiety might affect how each person reacts to surgical intervention and postoperative pain relief [3-4]. Preoperative anxiety may have an impact on a variety of postoperative outcomes, including worse quality of life and cognitive function, increased informational need, impaired memory and attention, prolonged hospitalization, depressed symptoms, and greater physical impairment [5]. Therefore, reducing preoperative patients' anxiety should be given top emphasis. Bibliotherapy, which is based on psychological interventions with a track record of success, might offer a more approachable source of psychological support. Instead of merely delivering facts, it employs a self-help book to direct and encourage patients to question problematic attitudes and actions, leading to enhanced self-management. The basic ideas of Cognitive Behavioral Therapy are included into bibliotherapy, which also offers exercises to help readers deal with their negative emotions [6]. A supplemental tool to a disease's clinical treatment is bibliotherapy. It is a technique that aids patients in coping with their circumstances by relating to the experiences of the characters in literature and developing their own tools via reading to make better health decisions and take charge of their lives and their sickness [7]. Numerous systematic reviews or meta-analyses have determined that bibliotherapy is effective in treating adult patients with emotional, physical, and mental health issues. Bibliotherapy was found to be considerably more successful than the control conditions in lowering the symptoms of depression or anxiety in both children and adults, according to a meta analysis of eight researches with a total of 979 participants [6]. An experimental study conducted on staff at a major medical centre found that a short, self-directed written course had exceptional short-term effectiveness for boosting resilience, mindfulness, and quality of life while lowering stress and anxiety [8]. According to a quasi-experimental study involving 84 moderately depressed adults, the aided self-help reading groups significantly reduced the participants' levels of depression when compared to the control group [9]. Nine original research studies that looked at bibliotherapy as a treatment to lessen the psychological effects of receiving a cancer diagnosis were found through a review of the literature analysis. Bibliotherapy is a viable and helpful supplementary therapy for cancer patients who are dealing with anxiety, depression, and inefficient coping, according to data synthesis from these researches [10]. The nursing professionals can contribute well to the reduction of anxiety among pre-operative patients, by non-pharmacological nursing interventions like bibliotherapy. Therefore it is of interest to investigate the efficacy of bibliotherapy among pre-operative adult patients.

## Methodology:

A quasi experimental research design was used to complete the study. A sample of 60 preoperative patients those who had been identified as having significant level of anxiety were selected for experimental group (30) and control group (30). The approach of non-probability purposive sampling was employed to choose the sample. The study was carried out in the following order;

E1 - Pre test level of anxiety in experimental group

C1 - Pre test level of anxiety in control group

X - Bibliotherapy (Only for experimental group)

E2 - Post test level of anxiety in experimental group

C2 - Post test level of anxiety in control group

Anxiety of patients assessed using the Hamilton Anxiety rating scale measurement. It consisted of 14 items. The Hamilton anxiety rating scale scores were ranged from 0 to 56. Each item was answered on a five point scale.

Items were scored as,

0 = Not present 

 1 = Mild 

2 = Moderate 

3 = Severe 

4 = Very severe 

In this study, the term "bibliotherapy" refers to a complementary therapy that involves giving preoperative patients comic books to read in order to lower their anxiety levels. Prior to surgery, bibliotherapy was given to the experimental group's sample twice daily for around 20 minutes. No intervention was given to the control group. The books were selected as per the sample's preferred language. On the previous day of surgery, the level of anxiety was measured again using Hamilton Anxiety rating scale for both control and experimental group. The collected data was analyzed and interpreted using various statistical methods.

## Results:

Majority of the sample belongs to the age group 40-49 years in both control and experimental group. Major percentage of sample was male patients. In experimental group majority was belongs to extended family and in control group major portion of sample was belongs to joint family. 30% of experimental group and 13% of control group has a history of hypertension. Table 1(see PDF) shows the evaluation of preoperative patients' anxiety levels in the experimental group and control group before to intervention. In the Experimental group, 0 (zero percent) reported mild anxiety during the pretest, 19 (63.34 percent) reported moderate anxiety, and 11 (36.66 percent) reported severe anxiety. In the Control group, 0 people experienced mild anxiety, 8 people had moderate anxiety (26.66 percentage), and 22 people had severe anxiety (73.34 percent). Table 2(see PDF) shows the evaluation of preoperative patients' anxiety levels in the experimental group and control group following intervention. In the Experimental group during the post-test, 17 (56.67%) people reported mild anxiety, 11 (36.67%) people reported moderate anxiety, and 2 (6.66%) people reported severe anxiety. In the Control group, there were no cases of mild anxiety, 12 cases of moderate anxiety, and 18 cases of severe anxiety. Figure 1(see PDF) show that, in pre test, the mean percentage score of experimental group was 80.10% and mean percentage score of control group was 85.66%. In post test, the mean score for experimental group was 50.66% and mean score of control group was 83.20%. Table 3(see PDF) represents, the post-test mean anxiety score for the Experimental group was 15.2 while it was 24.96 for the Control group. The predicted value was 9.94, and at p > 0.05, it is significant. It demonstrates that bibliotherapy was successful in lowering the anxiety level. So, it is agreed that study hypothesis (H1) is true.

## Discussion:

According to the current study, preoperative patients in the control and experimental groups both experienced moderate (45%) or severe (55%) anxiety. A comparison study of the severity of preoperative anxiety using 3 distinct scales lends support to this conclusion. According to the STAI, 44.4 percent, and 49.3 percent of participants in the study reported having anxiety, respectively. The majority of individuals exhibited moderate to high levels of preoperative anxiety, and the scales and instruments used to gauge this level in surgical patients could be used interchangeably [11]. Another cross-sectional study carried out in Jordan to determine the prevalence of preoperative anxiety among adult patients undergoing elective surgical procedures at a tertiary teaching hospital showed that the fear of Covid-19 in-hospital transmission made 36 (4.5 percent) of the total sample reluctant to undergo this surgery and 19 (7.9 percent) highly anxious patients (p = 0.002). This study showed that severe preoperative anxiety was present in 30.1% of patients, with worry about pain following surgery being the most frequent cause associated with anxiety on the day of operation [12]. An evaluation of preoperative anxiety, surgical anxiety, and anesthetic anxiety utilizing a cross-sectional single-center survey using the validated Amsterdam anxiety and information scale (APAIS) and modified numeric rating scale (mNRS). According to APAIS ratings, 92.6 percent of participants in the study exhibited preoperative anxiety. The typical APAIS anxiety score was 9.9 overall (SD 3.6). High anxiousness was indicated by 40.5 percent. The average level of anxiety was higher for surgery than for anaesthesia. As a result, more patients were significantly more fearful of surgery (score difference < 2) than of anaesthesia (642, 20.8 percent, 95 percent CI 19.4-22.3) (48, 1.6 percent, 95 percent CI 1.2-2.1) [13]. The purpose of the current study was to evaluate how well bibliotherapy reduced pre-operative patients' anxiety. The study's findings showed that the experimental group's mean percentage anxiety score at the pre-test was 80.10 percent, whereas the control group's mean percentage anxiety score was 85.66 percent. After the test, the experimental group's mean anxiety score was 50.66 percent, while the control group's mean anxiety score was 83.20 percent. It is evident that bibliotherapy was successful in lowering pre-operative patients' anxiety levels. An experimental study that looked at how bibliotherapy affected patients who had been recognized as having health issues lends weight to this conclusion. The 40 participants-patients from GP offices-were divided into two groups at random, one of which received bibliotherapy and the other did not. In half of the patients, a medical issue was present. Prior to and during the bibliotherapy intervention, which took the form of a cognitive-behavioral self-help booklet for people with health anxiety, anxiety levels were measured. At the post-test, patients in the bibliotherapy group displayed lower levels of anxiety, even when they were simultaneously dealing with a definite physical issue. These findings support the notion that self-help books can be a useful and practical intervention in cognitive behavioral therapy [14]. The mothers of children between the ages of 2 and 10 who were having tonsillectomy and/or adenoidectomy procedures participated in yet another quasi-experimental study. The study assessed the impact of reading a kid's book before surgery on pre- and postoperative anxiety and distress. The outcomes showed that the prep book can benefit children and parents before and after surgery by educating them and lowering their worry [15].

## Conclusions:

In order to promote mental health, bibliotherapy uses a number of materials, including self-help workbooks, pamphlets, novels, and audio books [10]. Data shows a significant effectiveness of bibliotherapy in reducing the anxiety level of pre-operative patients. Many number of studies revealed the existence of preoperative anxiety among patients either in moderate or in severe form. Nurses can effectively use this non-pharmacological intervention for reducing the anxiety of patients regarding surgery and thereby reducing the post operative complications. Various categories of books can be used for bibliotherapy according to the level of understanding of patients. This method can be applied in reduction of anxiety of patients in areas other than pre-operative situations also.
